# Risk factors for post-cerebral infarction cognitive dysfunction in older adults: a retrospective study

**DOI:** 10.1186/s12883-024-03574-7

**Published:** 2024-02-20

**Authors:** Fanyuan Ma, Qian Zhang, Jinke Li, Liping Wu, Hua Zhang

**Affiliations:** 1 Department of Geriatrics, Tangdu Hospital, Air Force Medical University, Xi’an, China; 2 Department of Geriatrics, Xijing Hospital, Air Force Medical University, Xi’an, China

**Keywords:** Cognitive dysfunction, Stroke, Type 2 diabetes, Cerebral infarction, Older adults, Risk factors

## Abstract

**Objective:**

Our research aims to elucidate the significance of type 2 diabetes (T2D) and provides an insight into a novel risk model for post-cerebral infarction cognitive dysfunction (PCICD).

**Methods:**

Our study recruited inpatients hospitalized with cerebral infarction in Xijing hospital, who underwent cognitive assessment of Mini-Mental State Examination (MMSE) from January 2010 to December 2021. Cognitive status was dichotomized into normal cognition and cognitive impairment. Collected data referred to Demographic Features, Clinical Diseases, scale tests, fluid biomarkers involving inflammation, coagulation function, hepatorenal function, lipid and glycemic management.

**Results:**

In our pooled dataset from 924 eligible patients, we included 353 in the final analysis (age range 65–91; 30.31% female). Multivariate logistic regression analysis was performed to show that Rural Areas (OR = 1.976, 95%CI = 1.111–3.515, *P* = 0.020), T2D (OR = 2.125, 95%CI = 1.267–3.563, *P* = 0.004), Direct Bilirubin (OR = 0.388, 95%CI = 0.196–0.769, *P* = 0.007), Severity of Dependence in terms of Barthel Index (OR = 1.708, 95%CI = 1.193–2.445, *P* = 0.003) that were independently associated with PCICD, constituting a model with optimal predictive efficiency.

**Conclusion:**

To the best of our knowledge, this study provides a practicable map of strategical predictors to robustly identify cognitive dysfunction at risk of post-cerebral infarction for clinicians in a broad sense. Of note, our findings support that the decline in serum direct bilirubin (DBil) concentration is linked to protecting cognitive function.

## Introduction

Cognitive dysfunction (CD), a major complication of cerebral infarction, appears to take negative effects on the five most commonly assessed domains: memory, attention, calculation, visuospatial function, and executive function [1]. Insufficient emphasis on CD has been posing a major burden to medical systems and impeding research progress, and there is a critical need for timely prevention. Many survivors exhibit cognitive deficits that endure or develop in the years following cerebral infarction [2], manifesting clinically as a stepwise or fluctuating deterioration in cognition. The cognitive domains that showed gradual decline after cerebral infarction, possibly through a synergistic interaction between neurodegeneration and cerebrovascular pathology [3].

There is growing recognition that people of all age groups with type 2 diabetes (T2D) develop progressively cognitive deficits that predominantly occur in older adults, and it is suspected that reduced cerebral vasoreactivity and altered resting cerebral blood flow may account for the lesser grade on cognitive tests [4]. The evidence as to whether T2D is associated with CD by various mechanisms and pathways is equivocal [5]. T2D is of the utmost clinical contributor to the overall burden of CD, and the need to sort out their relationship is a research priority.

Given that a variety of factors may initiate or accelerate CD in older adults, developing accurate prediction models is of paramount significance. Hence, our study specifically aims to explore significant risk factors with predictive value for post-cerebral infarction CD diagnosis outcome. Identifying outcome determinants could offer clinical insights into cognitive decline, as well as improve prevention strategies considered suitable for clinical use.

## Methods

### Participants

Participants were older adults aged 65 and above hospitalized from January 2010 to December 2021. To be enrolled, participants had to be diagnosed as cerebral infarction and filled out Mini-mental State Examination (MMSE) for cognitive assessment. We extracted data using an electronic miniature form in Xijing Hospital affiliated to Air Force Medical University. Data on demographic characteristics, fluid biomarkers, and cardiac-cerebral vascular diseases were collected from medical records.

Participants were excluded if they had any of the following: (1) under the age of 65; (2) missing data of MMSE score; (3) Dementia, Alzheimer’s disease, Binswanger’s disease; (4) unclear diagnosis as Type 2 Diabtes; (5) malignant or undermined tumor; (6) psychonosema, alcohol dependence.

### Risk or protective factors

Besides demographic characteristics (e.g., Gender, living area, Alcohol consumption, Education level), fluid biomarkers combining peripheral blood cells, coagulation function, hepatorenal function, Homocysteine (HCY) and Glycated hemoglobin (HbA_1c_) were involved. Scale tests refered to MMSE scale, Hamilton Anxiety Scale (HAMA), Hamilton Depression Scale (HAMD), and Barthel Index (BI).

Education level is used as a ranked variable, differing from illiteracy, elementary school, junior high school, and above, in terms of application conditions of MMSE. Amended evaluations to verify CD are as follows: those who scored < 17 at the illiteracy level; those scored < 20 at the elementary school level; those who scored < 24 at junior high school and above [6]. Degree of dependence is also used as a ranked variable, varying from independent, mildly dependent, moderately dependent to severely dependent, according to the scores patients have gained [7, 8].

### Statistical methods

Kolmogorov-Smirnov test was used to test the normality of continuous variables. Group comparisons were performed using the one-way analysis of variance (ANOVA) (parametric variables) or Mann-Whitney U test (non-parametric variables) as appropriate. Mean ± standard deviation values applied to quantitative data that was normally distributed,and the median (quartile), i.e., M (P_25_-P_75_), applied to the quantitative data that was non-normally distributed. Categorical variables were compared using a chi-square test. Multivariate logistic regression models went for significantly risk factors that might be related to cognitive impairment. Significance was set for all comparisons at *P* < 0.05.

## Results

**Fig. 1:**
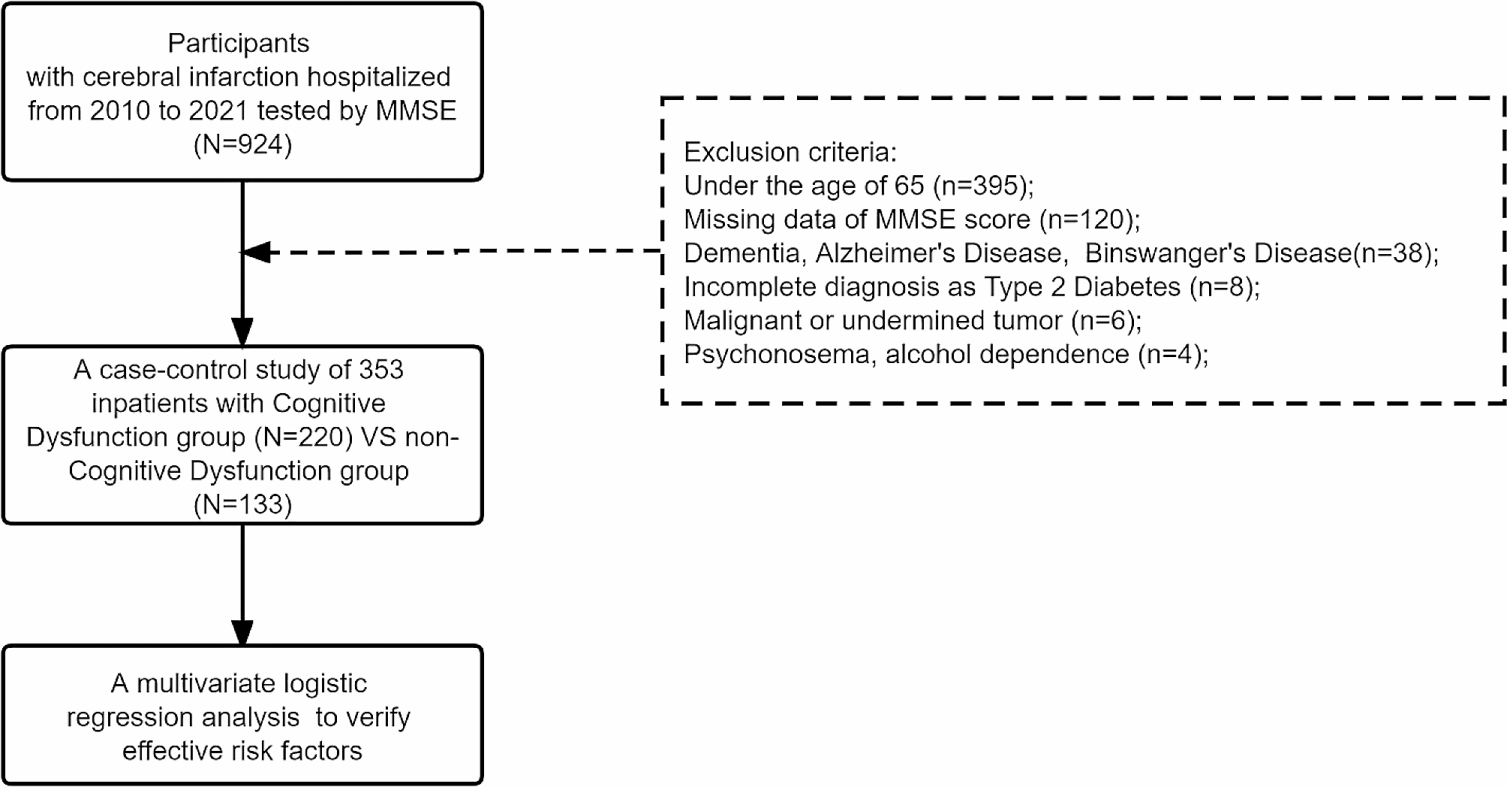
The flow chart and exclusion criteria of the retrospective study

Of the 924 participants, a total of 353 patients were included in the final analysis (Shown in Fig. 1). In the total of the study samples, the mean age was 73 years (SD = 5.708) and 246 patients (69.69%) were men. 99 patients (28.05%) lived in rural areas. Our retrospective trial examined the effects of multidomain fluid biomarker that simultaneously targeted blood pressure, lipid and glycemic control, inflammation and coagulation, physical inactivity, and hepatorenal function.

220 patients were diagnosed with CD, based on their performance on MMSE scores standardized by education level. The remaining 133 patients were diagnosed with non-cognitive dysfunction (NCD). No significant differences were detected in age, gender, education level, current smoking and drinking (Shown in Table 1). As for common complications of old people, the presence of T2D appeared to be positively associated with incident CD. However, no significant difference was found between the two groups in HbA_1c_. On the other hand, interestingly, elevated level of bilirubin tends to be negatively associated with incident CD, seemingly playing a protective role.

**Table 1:** Demographic and clinical characteristics of patients stratified according to cognitive dysfunction

Characteristics		NCD group (*n* = 133)	CD group (*n* = 220)	F/χ^2^/Z	P value
Demographic Features and Clinical Diseases	Age (y)	72 (67, 76)	73 (68, 76)	0.932	0.351
	Gender, male, n (%)	94 (70.68%)	152 (69.09%)	0.099	0.753
	Area, rural, n (%)	28 (21.05%)	71 (32.27%)	5.171	**0.023** ^*^
	Alcohol comsumption, n (%)	21 (15.79%)	54 (24.55%)	3.798	0.051
	Smoking, n (%)	53 (39.85%)	87 (39.55%)	0.003	0.955
	Education level			−0.353	0.724
	Illiteracy, n (%)	3 (2.25%)	13 (5.91%)		
	Elementary school, n (%)	31 (23.31%)	45 (20.45%)		
	Junior high school & above, n (%)	99 (74.44%)	162 (73.64%)		
	Hypertension, n (%)	101 (75.94%)	162 (73.64%)	0.232	0.630
	Coronary Artery Disease, n (%)	26 (19.55%)	43 (19.55%)	0.000	0.999
	Cerebral Arterial Stenosis, n (%)	19 (14.29%)	23 (10.45%)	1.161	0.281
Scale Tests	MMSE score	25 (25, 27)	19 (15, 22)	−14.595	**0.000** ^*^
	HAMA score	9 (7,13)	11 (7, 15)	1.588	0.112
	HAMD score	10 (6, 15)	10 (7, 16)	0.366	0.714
	Severity of dependence (BI)			−4.117	**0.000** ^*^
	Independent, n(%)	60 (45.11%)	54 (24.55%)		
	Mildly dependent, n(%)	60 (45.11%)	125 (56.82%)		
	Moderately dependent, n(%)	12 (9.23%)	32 (14.54%)		
	Severely dependent, n(%)	1 (0.75%)	9 (4.09%)		
Peripheral Blood Cells & Coagulation Function	White blood cell (×10^9^/L)	5.78 (4.83, 7.18)	5.77 (4.88, 6.90)	−0.438	0.662
	Neutrophil %	0.613 ± 0.098	0.605 ± 0.099	0.288	0.462
	Lymphocyte %	0.289 ± 0.091	0.290 ± 0.090	0.262	0.905
	Neutrophil (×10^9^/L)	3.60 (2.77, 4.56)	3.54 (2.65, 4.35)	−0.719	0.472
	Lymphocyte (×10^9^/L)	1.65 (1.28, 2.02)	1.61 (1.28, 2.09)	−0.304	0.761
	Blood platelet (×10^9^/L)	181 (149, 207)	184 (152, 218)	0.935	0.350
	PT (s)	11.00 (10.40, 11.40)	11.00 (10.50, 11.50)	0.196	0.845
	APTT (s)	25.80 (23.00, 28.40)	25.90 (23.40, 28.75)	0.499	0.617
	Fibrinogen (g/L)	2.73 (2.35, 3.23)	2.90 (2.39, 3.40)	1.297	0.195
	Thrombin time (s)	18.20 (17.30, 19.00)	18.10 (17.25, 19.00)	−0.354	0.724
	D-dimer (mg/L)	0.36 (0.24, 0.70)	0.40 (0.24, 0.66)	0.719	0.472
	FDP (µg/ml)	1.85 (1.40, 2.76)	1.95 (1.43, 2.52)	0.285	0.775
	PT% (%)	94.40 (85.60, 100.00)	94.30 (85.65, 104.00)	0.846	0.398
	INR	0.97 (0.92, 1.03)	0.97 (0.92, 1.02)	−0.019	0.985
Hepatorenal Function	ALT (IU/L)	20 (15, 28)	19 (14, 25)	−1.793	0.073
	AST (IU/L)	20 (16, 25)	19 (15, 23)	−1.906	0.057
	γ-GGT (IU/L)	20 (15, 30)	23 (16, 35)	1.673	0.094
	Totel protein (g/L)	66.0 ± 5.8	64.8 ± 5.5	0.345	0.054
	Globulin (g/L)	26.1 ± 4.3	26.1 ± 4.1	0.224	0.921
	Albumin (g/L)	39.9 ± 3.4	38.7 ± 3.5	0.349	**0.003** ^*^
	Total bilirubin (µmol/L)	13.1 (10.1, 15.9)	11.1 (8.8, 14.9)	−2.993	**0.003** ^*^
	Direct bilirubin (µmol/L)	4.7 (3.4, 6.3)	4.0 (3.1, 5.4)	−2.307	**0.021** ^*^
	Indirect bilirubin (µmol/L)	8.5 (6.3, 10.0)	7.1 (5.5, 9.4)	−2.863	**0.004** ^*^
	Alkaline phosphatase (IU/L)	73 (61, 86)	76 (65, 92)	1.792	0.073
	Cystatin-C (mg/L)	1.11 (0.98, 1.22)	1.10 (0.98, 1.28)	0.325	0.745
	Blood urea nitrogen (mmol/L)	5.09 (4.29, 6.51)	5.30 (4.35, 6.73)	0.922	0.357
	Serum creatinine (µmol/L)	87 (74, 100)	87 (72, 100)	−0.051	0.959
	Serum uric acid (µmol/L)	288 (228, 345)	270 (230, 323)	−1.775	0.076
BP, lipid, weight and glycemic management	BMI (kg/m^2^)	24.3 (23.1, 26.1)	24.0 (22.6, 26.0)	−0.745	0.456
	Systolic blood pressure (mmHg)	139 (131, 156)	140 (130, 151)	−0.599	0.549
	Diastolic blood pressure (mmHg)	80 (72, 87)	79 (72, 85)	−0.596	0.551
	T2D, n (%)	45 (33.83%)	108 (49.09%)	7.856	**0.005** ^*^
	HbA_1c_ (%)	6.4 (5.6, 7.4)	6.6 (5.9, 7.9)	1.796	0.073
	Blood glucose (mmol/L)	7.4 (6.1, 9.4)	7.4 (6.0, 10.8)	0.403	0.687
	Total cholesterol (mmol/L)	3.67 (3.05, 4.26)	3.65 (3.07, 4.43)	0.138	0.890
	Triglyceride (mmol/L)	1.15 (0.90, 1.60)	1.18 (0.92, 1.69)	0.744	0.457
	HDL-C (mmol/L)	1.07 (0.89, 1.28)	1.06 (0.90, 1.22)	−0.340	0.734
	LDL-C (mmol/L)	2.00 (1.49, 2.60)	2.11 (1.56, 1.76)	0.647	0.518
	Apolipoprotein-A1 (g/L)	1.08 (0.93, 1.29)	1.08 (0.96, 1.22)	−0.160	0.873
	Apolipoprotein-B (g/L)	0.66 (0.51, 0.79)	0.65 (0.54, 0.84)	1.018	0.309
	HCY (µmol/L)	13.33 (10.37, 17.48)	12.85 (10.50, 17.24)	−0.022	0.983
	Vit B_12_ (pmol/L)	262 (176, 510)	331 (211, 549)	1.926	0.054
	Folic Acid (nmol/L)	13.99 (8.62, 22.32)	12.65 (8.39, 19.60)	−0.988	0.323

^∗^*P* < 0.05

*Abbreviation* PT = prothrombin time; APTT = activated partial thromboplastin time; FDP = fibrinogen degradation products; INR = international normalized ratio; GGT = glutamyl transpeptidase; HDL-C = high density lipoprotein-cholesterol; LDL-C = low density lipoprotein-cholesterol; HCY = Homocysteine

**Fig. 2:**
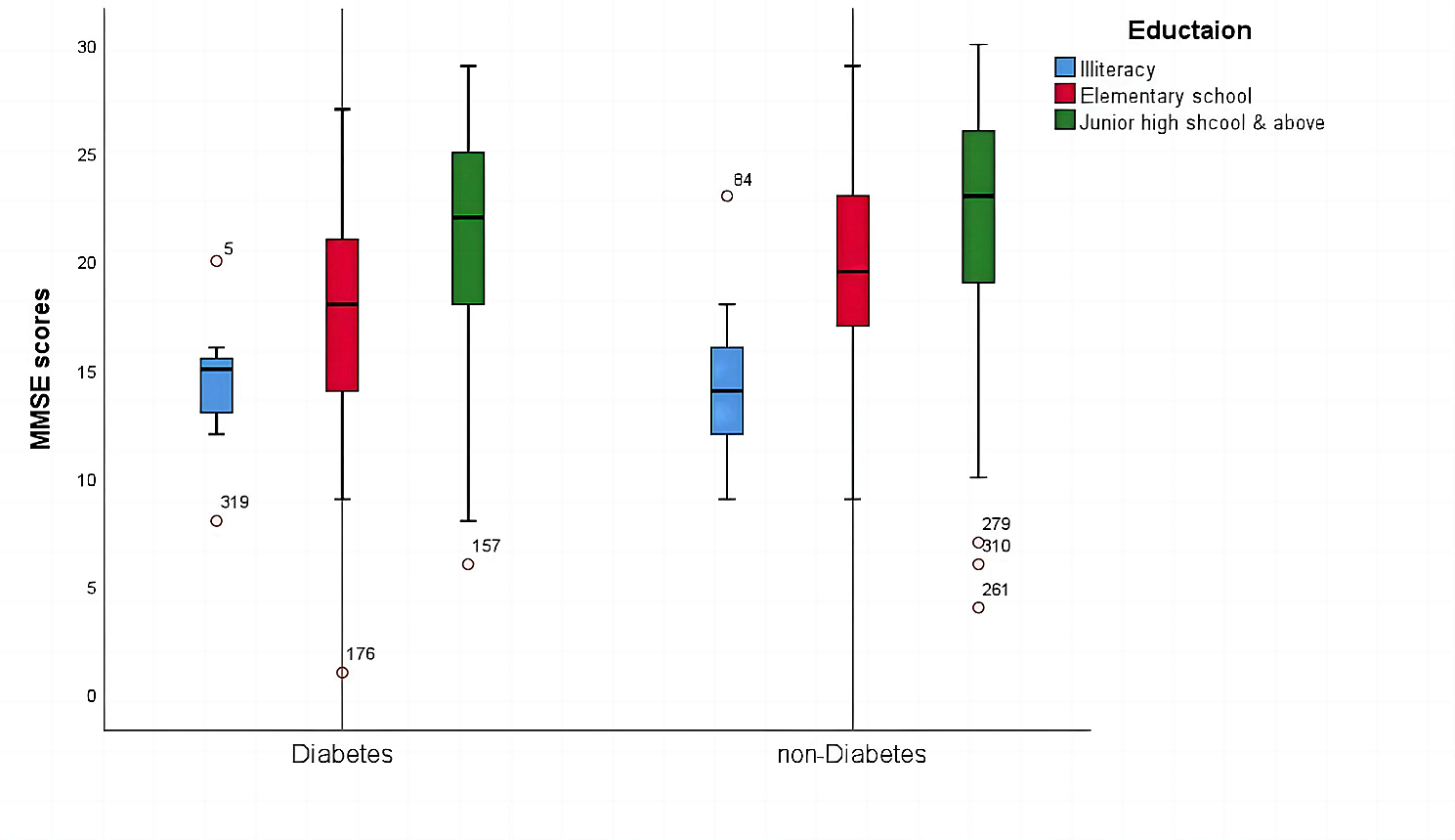
The box plot to show the correlations between T2D and MMSE scores on account of education level

To further explore effective risk factors for the post-cerebral infarction cognitive dysfunction (PCICD) on the whole, a pooled logistic regression analysis of the available data after adjusting many confounding factors including age, gender, and education level (all *P* < 0.05), suggests that living in rural areas leads to a 1.976-fold increased risk of PCICD (OR = 1.976, 95%CI = 1.111–3.515, *P* = 0.020). With respect to complications, incident T2D confers an increase in risk (OR = 2.125, 95%CI = 1.267–3.563, *P* = 0.004). The risk of post-cerebral infarction with hyperbilirubinemia is 0.388 times lower compared to normal range of bilirubin for CD (OR = 0.388, 95%CI = 0.196–0.769, *P* = 0.007). Barthel Index is presumably associated with PCICD because it reflects physical functional limitations or even the greater dependency severity (OR = 1.708, 95%CI = 1.193–2.445, *P* = 0.003) (Shown in Table 2). In addition, CD may occur as a result of severe dependency levels. Our study found that cerebral infarction survivors with T2D, compared to those without, experienced a faster decline in cognition scores at baseline (Shown in Fig. 2).

**Table 2:** Logistic regression analysis to verify risk factors of incident cognitive dysfunction

	Regression coefficient (β)	Standard error (SE)	Wald	P value	Odds ratio (OR)	95% confidence interval (CI)
T2D	0.754	0.264	8.165	**0.004** ^*^	2.125	1.267–3.563
Area (Rural)	0.681	0.294	5.381	**0.020** ^*^	1.976	1.111–3.515
Dependence (BI)	0.535	0.183	8.560	**0.003** ^*^	1.708	1.193–2.445
D Bil	-0.946	0.349	7.358	**0.007** ^*^	0.388	0.196–0.769
Age	0.014	0.019	0.476	0.490	1.014	0.976–1.053
Gender (male)	-0.034	0.248	0.018	0.892	0.967	0.594–1.573
Education level	-0.139	0.211	0.434	0.510	0.870	0.576–1.315

^∗^*P* < 0.05

**Fig. 3:**
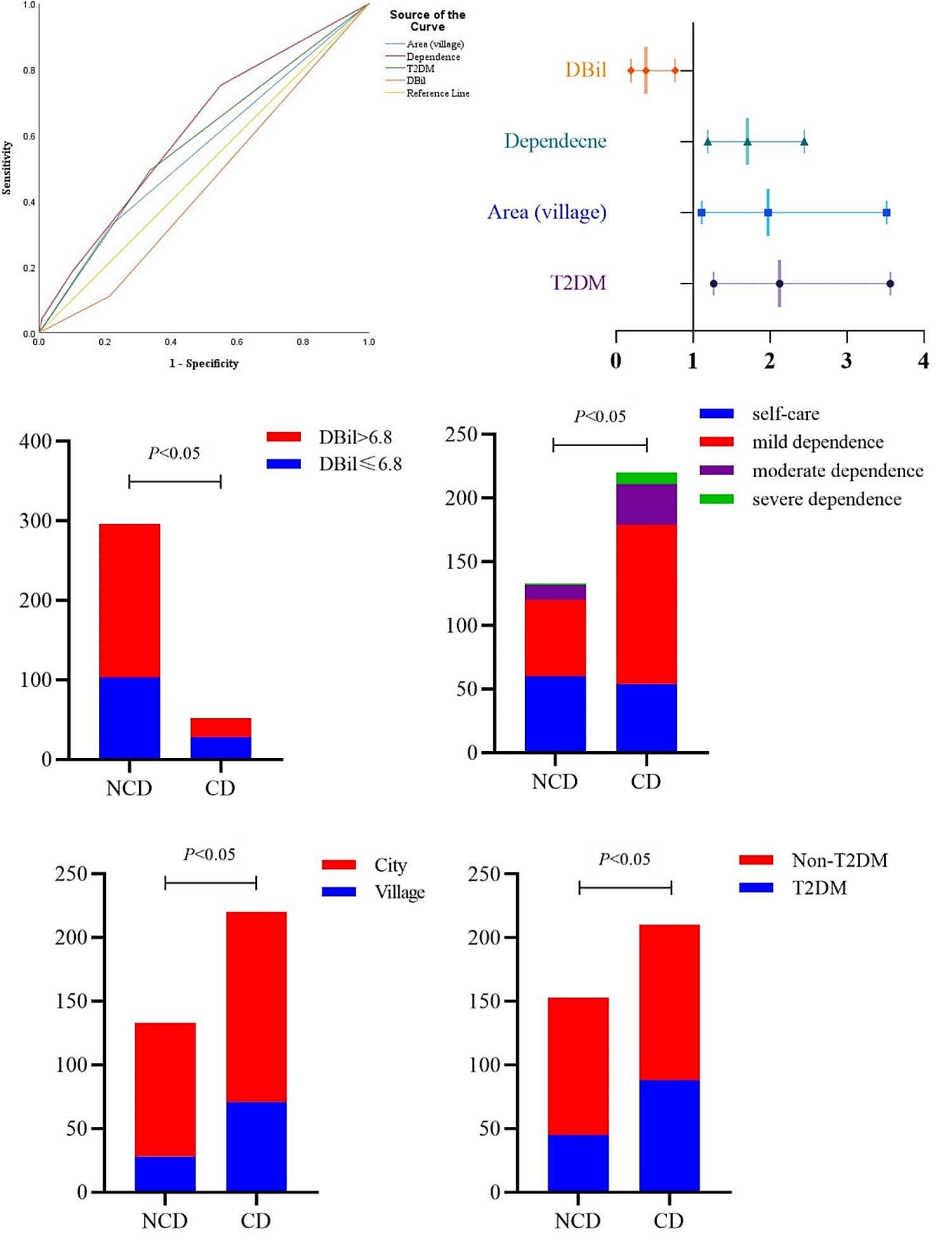
ROC curve and Forest Plot to show risk factors for incident post-cerebral infarction cognitive dysfunction. It is obvious that DBil plays a protective role. The contingency tables showed the four risk factors distribution in the evaluation the logistic model

Some limitations of our study should be noted. Post-cerebral infarction cognitive trajectories might be time-variant, as such, leading to inaccuracy in the timing of assessment due to inconsistent estimates. we have created a multifactor model that reasonably predicts PCICD following cerebral infarction (Shown in Fig. 3), and the contingency tables could demonstrate the distribution of the four risk factors following a logistic regression assignment. It is quite clear that elevated DBil level plays a protective role in CD, and the effects of bilirubin may offer novel insights into better informed prevention strategies and follow-up.

## Discussion

While the interactions are being addressed by ongoing massive research efforts, CD following a cerebral infarction event is principally attributed to direct damage to brain regions essential for cognition [9], cerebral structural lesion, and disrupted connectivity [10], neurotoxicity, oxidative stress injury and metabolic disturbances [11]. Some factors, mainly age, are also known to contribute to vascular remodeling and angiogenesis being attenuated in the aging brain, which results in accelerated cognitive decline. People with T2D in all age groups develop subtle cognitive changes that are aggravated with time prolonging, primarily affecting older adults [4]. However, no overwhelming evidence indicates that T2D does indeed accelerate the rate of cognitive aging [12]. Even then, adults aged 65 years or older should be screened for early detection of CD annually as appropriate [13].

Our study validates a practicable prediction model for PCICD, which in some respects showed an inextricable link between T2D and cognition, but not necessarily implying causality. The final model, consisting of rural areas, the presence of T2D, bilirubin, and Barthel index, allows for viable clinical implementation due to its simplicity, although waiting further testing in prospective studies. We envision that may be conductive to prioritizing patients for cognitive assessment where resources are restricted. Although our model is formulated in view of older adults aged 65, living in rural areas, which we were not able to control for, seems of particular importance here. We do not claim that our model is definitive, but it allows to highlight the complex of incident CD. Our research highlights the need for consideration of common comorbidities, simultaneous and interactive.

Based on data from a real-world sample, the results of our study supported the impelling role of T2D on cognitive deterioration, perhaps through diabetes-derived endothelial dysfunction and microvessel damage [14]. Although more attention shall be paid, greater glucose variability in the early phase post-cerebral infarction has been associated with CD [15]. It is suspected whether there are positive effects on cognition is bound up with the absence of a concurrent history of diabetes and hypertension [16]. Whereas, no significant difference is found in the HbA_1c_. The correlation between HbA_1c_ levels and cognition remains disputed [17].

Survivors of cerebral infarction may be challenged not only with cognitive consequences, but also with physical inactivity [18]. Barthel index is used extensively, assessing functional status to identify older adults who need higher levels of nursing assistance [19]. The Combination of motor and cognition impairment is relatively common among stroke survivors, associated with executive function and global cognition [20]. The role of cognition in functional recovery remains a benchmark of in cerebral infarction [21]. A validated measure of the motor-cognitive interval shall be necessary to predict incident CD in individuals with history of cerebral infarction.

It is speculated that there may be a close tie between plasma bilirubin and cerebral infarction. By contrast, few studies assessed the predictive value of bilirubin for CD as a clinical outcome, which requires a multifaceted analytical approach. As per our analysis, the association even tends to be a reverse where bilirubin is not accounted for or is taken as a protector of cognitive dysfunction, which also implies the possible benefits of elevated bilirubin, echoing a prior study where bilirubin levels were found to be positive correlated with working memory [22]. In concordance with the previous study [23], as the inverse relationship is revealed, our study suggests the protective effect of bilirubin on antioxidant and anti-inflammatory properties is considered an excellent potential marker. Nevertheless, another study declared serum TBil concentration was positively correlated with immediate memory [24], inconsistent with our results. It is suspected the memory impairment may be attributed to deficiency of the antioxidant system caused by decreased bilirubin [22]. It is thus significant for future studies to disentangle the potential effects of hyperbilirubinemia on PCICD. Due to the limited number of included cases, we can not find evidence in this regard suggesting the associations of hyperbilirubinemia and PCICD differed by potential heterogeneity sources, including subtype, duration of disease and time point of assessment. As such, bilirubin is likely to emerge as a credible risk and prognostic parameter serving to augment clinical assessment.

## Conclusion

Taken together, the diagnosis and prediction of PCICD by biomarkers has become a hot research topic, risk factors involving degree of dependence, living area, the presence of T2D, and bilirubinemia. To the best of our knowledge, this study provides a practicable map of strategical predictors to robustly identify CD at risk of post-cerebral infarction for clinicians in a broad sense. Of note, our findings support that the decline in serum DBil concentration is linked to protecting cognitive function. In order to develop more accurate models for predicting cognitive outcomes after cerebral infarction, future researches should consider the possibility of interactions between multiple pathways of influence and predictors.

## Data Availability

The datasets used and/or analysed during the current study available from the corresponding author on reasonable request.
